# Snack Consumption Patterns among Canadians

**DOI:** 10.3390/nu11051152

**Published:** 2019-05-23

**Authors:** Hassan Vatanparast, Naorin Islam, Rashmi Prakash Patil, Mojtaba Shafiee, Jessica Smith, Susan Whiting

**Affiliations:** 1 College of Pharmacy and Nutrition, School of Public Health, University of Saskatchewan, Saskatoon, SK S7N 4Z2, Canada; naorin.islam@usask.ca (N.I.); rashmi.patil@usask.ca (R.P.P.); mos866@usask.ca (M.S.); susan.whiting@usask.ca (S.W.); 2 Bell Institute of Health and Nutrition, General Mills, Minneapolis, MN 55427-3870, USA; Jessica.Smith@genmills.com

**Keywords:** snack, nutrient intake, food occasions, snacking patterns, food choices, body mass index, national survey, Canadian population

## Abstract

The snacking prevalence, frequency of daily snack consumption, and the contribution of snacks to daily energy intake have substantially increased globally. The aim of this study was to examine the patterns of snack consumption among a representative sample of Canadians aged 2 and older. Nationally representative dietary data from the Canadian Community Health Survey (CCHS) conducted in 2015 (*n* = 19,677 participants aged ≥2 years) were used to describe snacking patterns. In all, 80.4% of Canadians reported consuming at least one snack per day, which varied between different age groups from 77.0% (≥55 years) to 96.4% (2–5 years). About 37% of snack consumers reported only one snack episode per day but nearly 10% reported four or more episodes of snacking. Snacking contributed to nearly 23% of total daily energy intake in Canadians, which was highest among younger children (27%) and lowest among older adults (20.8%). There were no significant differences in obesity measures comparing snack consumers and non-consumers in children and adults. Snacking considerably contributes to total nutrient and energy intake of Canadians. Promoting nutrient-dense snacks provides an opportunity to improve overall diet quality.

## 1. Background

In most societies, people usually obtain the majority of their energy and nutrient requirements from three planned meals (breakfast, lunch, and dinner), which are often consumed with family, friends, colleagues, etc. at relatively predictable times and in dedicated places [1]. All other eating occasions occurring outside the context of main meals are considered as “snacks”, which are different from regular meals in terms of nutritional profile, time of consumption, and frequency of consumption [1,2]. Although some studies suggest a link between snack consumption and some chronic health conditions, especially overweight and obesity [3,4,5,6], others have found evidence that snacking on healthy foods such as whole fruits and vegetables are associated with better overall diet quality and has no impact on body weight [6,7]. Moreover, there are reports suggesting that skipping snacks may have some detrimental effects on human health. In this regard, Maugeri et al. reported that skipping an afternoon snack is associated with a higher risk of poor cardiovascular health, after adjustment for well-known risk factors [8].

Recent studies suggest that total energy intake has increased over the past few decades, with shifts away from meals to snacks [9,10]. The snacking prevalence, the frequency of daily snack consumption, and the contribution of snacks to daily energy intake have substantially increased in all age groups in different parts of the world [11,12,13]. Surveys conducted in the United States revealed that the prevalence of snack consumption over a 2-day period has dramatically increased among American adults from 71% in 1977 to 97% in 2003–2006. Moreover, over this time period, there has been a significant increase in the frequency of consumption (from 1.26 to 2.23 snacks/day) and the contribution of snacks to total energy intake (from 18% to 24%) [13]. Additionally, a comparison between data collected through the National Diet and Nutrition Survey (NDNS) and data from a Northern Irish cohort of adolescents demonstrated significant increases in the percentage of energy intake from snacks (29.8% in 1997 vs. 32.5% in 2005), the portion size consumed, and the frequency of snack consumption over an 8-year period [12]. Similar trends were also observed in Australia, where the percentage of children and adolescents snacking (92.5% in 1995 vs. 95.8 % in 2011–2012), mean number of snacking occasions (2.0 in 1995 vs. 2.5 in 2011–2012), and the energy contribution from snacking (24.1% in 1995 vs. 30.5% in 2011–2012) increased over time [11].

Current data regarding the patterns of snacking among the Canadian population are limited [14]. Therefore, using nationally representative data from the 2015 Canadian Community Health Survey (CCHS), the objective of the present study was (a) to examine patterns in snacking behavior, percent energy, and nutrient intake from snacking and the number of daily snacking occasions among participants with different age groups; and (b) to describe the socio-economic and lifestyle characteristics of individuals in relation to snack consumption.

## 2. Subject and Method

### 2.1. Data Source, Study Design, and Dietary Data Collection

This study was based on the Canadian Community Health Survey Data (CCHS) Nutrition 2015, a cross-sectional survey conducted by Statistics Canada. This survey included all individuals who were one year or older living in private dwellings in the 10 Canadian provinces. The respondents reported their food and beverage consumption including types and amounts of foods consumed, eating occasion (e.g., breakfast, lunch, and snack), and time of consumption using a computer-assisted dietary recall method known as the Automated Multiple Pass Method (AMPM) [15]. Children aged 1 to 6 years old participated in the survey by proxy interview, children aged 6 to 12 years old participated with parental guidance, and individuals 12 years and older participated by the non-proxy method [16]. Permission to access and conduct the analyses of 2015 data were obtained from the Research Data Center Program of Statistics Canada. We followed the vetting regulation set by Statistics Canada in releasing the results of the study.

### 2.2. Analytical Sample

This study included 19,677 respondents representing the Canadian population. Children less than 2 years, pregnant women, lactating women, individuals who did not report any food intake in the 24-h recall, individuals who reported daily caloric intake outside of the range of 200–8000 kcal, and individuals with extreme positively high intakes of nutrients were excluded from this study.

### 2.3. Snack Consumption

Individuals self-identified the type of eating occasion on their 24-h recall. Any individual who reported consuming a “snack” as a food occasion on day 1 of the 24-h recall was defined as a snack consumer. Breakfast/brunch, lunch and dinner consumers were defined in the same way. In this study, 16,179 individuals reported snack consumption. Descriptive analysis included the prevalence of snack consumption by five age groups (2–5 years (younger children), 6–12 years (children), 13–18 years (adolescents), 19–54 years (adults) and ≥55 years (older adults)), sex, and frequency of snacking. Sociodemographic differences between snack consumers and non-consumers were classified within two age groups, 2–18 years and ≥19 years.

### 2.4. Daily Nutrients and Food Groups Intake

The Automated Multiple Pass Method (AMPM) uses the Canadian Nutrient File data on food items and the respected energy and nutrient amounts [14]. The contribution of snacks to the daily intake of nutrients and energy was calculated for the five age groups. The contribution of four meal occasions (snack, breakfast/brunch, lunch, and dinner) to total daily intake of nutrients and energy was also calculated for all age groups. Furthermore, we obtained the distribution of food groups consumed at each meal occasion. The five main food groups were defined based on the Canada Food Guide 2007 [17]: vegetables and fruit, grain products, milk and alternatives, meat and alternatives, and other food. The percent contribution of whole grain and non-whole grain foods (enriched and not enriched) to total grain intake of snacks were also calculated. Since the analyses began before the release of the new Canada Food Guide (2019) [18], we followed the Canada Food Guide (2007) [16] for food group analyses. Percentages of food groups consumption at various meal occasions were also calculated among snack consumers. The food group consumption was measured using serving sizes.

### 2.5. Other Variables of Interest

The body mass index (BMI) z-score calculated for children 5 to 19 years followed the guidelines of the World Health Organization (WHO) (version 3.2.2, January 2011) [19]. For adults who were 19 years and over, the existing CCHS 2015 variable on measured BMI variable was used. Other categorical sociodemographic variables included sex, smoking (yes, no), ethnicity (white, nonwhite), education (university degree, lower than university degree), marital status (yes, no), food security (secure, insecure), overweight/obese (yes, no), residence (urban, rural), and immigrant (yes, no).

### 2.6. Statistical Analyses

Data are presented as percentages and standard error (SE) or as mean and SE. All statistical analyses were carried out using SAS, version 9.4 (SAS Institute) at Sky Research Data Center, University of Saskatchewan. To produce the population level estimates, appropriate weighting and bootstrapping procedures were applied as per Statistics Canada’s recommendations [20]. We used the chi-squared test to compare the distribution of categorical sociodemographic variables between snack consumers and non-consumers. Analysis of covariance (ANCOVA) was used to compare the proportions of nutrient contributions from snacks to daily intakes among the five age groups. A similar test had been applied to obtain differences in daily nutrient intake between snack consumers and non-consumers. For all analyses, alpha was set at 0.05 for statistical significance.

## 3. Results

Overall, 80.4% of Canadians reported consuming a snack on any given day in 2015. Among snack consumers, 49.6% ± 0.4 were males and 50.3% ± 0.4 were females. The prevalence of snack consumption when studied further, was highest among children aged 2–5 years (96.4 ± 0.9), followed by children aged 6–12 years (92.9 ± 1.0), adolescents aged 13-18 years (85.3 ± 1.1), adults aged 19–54 years (78.7 ± 1.1), and lastly among older adults ≥55 years (77.0 ± 0.9). During the 24-h period, the frequency of snack consumption was observed to be the highest between 15:00 p.m. and 20:00 p.m. at 36.3% ± 0.7, followed by 31.2% ± 0.7 between 20:00 p.m. and 12:00 midnight, and 24.4% between 10:00 a.m. and 15:00 p.m. The lowest frequency of snack consumption (8.1%) occurred between 12:00 midnight and 10:00 a.m.

### 3.1. Prevalence of Snack Consumption

Table 1 displays data on sociodemographic characteristics of snack consumers and non-consumers among children and adults separately. Among children, snack consumers were more likely to be younger by age and to be Caucasian. Among adults, snack consumers were more likely to be younger, Caucasian, and more highly educated. Adult snack consumers had a mean BMI of 27.3 ± 0.1, and non-consumers had a mean BMI of 27.7 ± 0.2, and children snack consumers had a mean BMI z-score of 0.46 ± 0.04 and non-consumers had a mean BMI z-score of 0.44 ± 0.1, without significant differences.

### 3.2. The Frequency of Snack Consumption among Canadians

**Figure 1:**
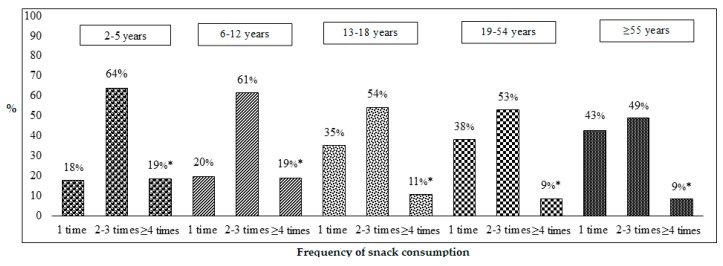
The frequency of snack consumption (%) among Canadians for five age groups ^1^. Data source: 2015 Canadian Community Health Survey–Nutrition. * Significant difference in the number of occasions of snack consumption within the specific age group. ^1^ All data were weighted and bootstrapped to the Canadian population. 2–5 years: *n* = 1,181,823; 6–12 years: *n* = 2,407,637; 13–18 years: *n* = 2,228,356; 19–54 years: *n* = 12,768,195; 55+ years: n = 8,310,789.

The frequency of snack consumption is presented based on the number of times the participants reported consuming snacks on a given day. Overall, 37% ± 0.8 of snack consumers reported consuming only one snack per day, while 53% ± 0.8 reported 2–3 snacking episodes per day, and 10% ± 0.5 reported 4 or more snacking episodes. Figure 1 displays the frequency of snack distribution by the five age groups with the frequency of consuming only one snack per day increasing from children to older adults.

### 3.3. Distributions of Food Group Consumption across Meal Occasions (Snack, Breakfast/Brunch, Lunch and Dinner)

**Figure 2:**
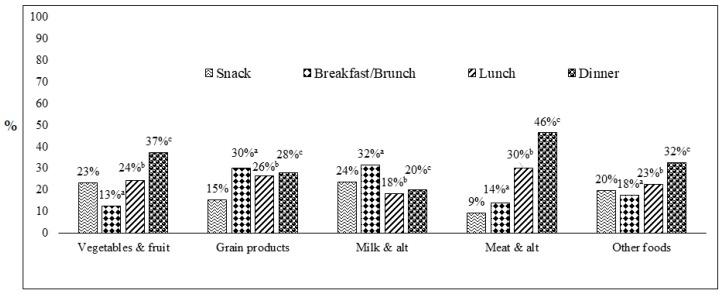
Percentages of food group consumption at various meal occasions (*n* = 26,896,800) (i.e., snack, breakfast/brunch, lunch, dinner) among snack consumers ^1^. Data source: 2015 Canadian Community Health Survey (CCHS)–Nutrition. CCHS 2015 dataset contains reports on meal occasions. The above meal occasions were exclusively analyzed as they fit the scope of the current study. ^1^ All data were weighted and bootstrapped to the Canadian population. a = significant difference between snack and breakfast/brunch consumption, b = significant difference between snack and lunch consumption, c = significant difference between snack and dinner consumption at 5% level of significance.

Figure 2 presents the distribution of food groups consumed, based on the Canada Food Guide (2007) [17], on various meal occasions. Distribution of the five food groups including vegetables and fruits, milk and alternatives, meat and alternatives, grain products, and other food group are noted. Vegetables and fruits consumption was the highest at dinner followed by snack and other meal occasions. Similar patterns of reporting were noticed for other food groups under the study, except for grain products and milk and alternatives.

### 3.4. Nutrient Contribution (%) of Snack, Breakfast/Brunch, Lunch, and Dinner to Daily Intake

Table 2 represents the distribution of nutrient contributions to daily intake from meal occasions. Snacking contributed to 22.7% of total daily energy intake in the Canadian population. In terms of macronutrients, snacking accounted for 25.8% of total carbohydrate intake, 23.4% of total fat intake, and 14.7% of total protein intake. The contribution to daily vitamin intake from snacking ranged from 13.4% for vitamin B_12_ to 23.4% for vitamin C. Moreover, the contribution of snacking to daily mineral intake in Canadians was the highest for potassium (20.7%) and the lowest for sodium (14.8%).

### 3.5. Energy and Nutrient Contribution of Snack among Five Age Groups

Energy and nutrient contribution from snacks to daily intake among children and adults (five age groups), is presented in Table 3. A significant difference in nutrient contributions was noted between all five age groups, for almost all the nutrients. The contribution of snacks to total daily energy intake was the highest among younger children (27.0%) and the lowest among older adults (20.8%). Among all age groups, the percent contribution of snacking to daily vitamin intake was highest for vitamin C (20.5–30.2%). The percent contribution of snacking to daily mineral intake was highest for calcium among younger children and older adults, for magnesium among adolescents and adults, and for potassium among older children.

### 3.6. The Contribution of Grain Subgroups to Total Grain Product Consumed as Snack

Table 4 displays data on the distribution (%) of whole grains, and non-whole grains (enriched and not enriched) at snack occasions. The three subgroups of grain products in this study are based on the Canada Food Guide (2007) [17]. For all Canadians, enriched non-whole grain was the highest contributor (66.6% ± 1.0) to total grain product intake as a snack, followed by non-whole grain not enriched (18.2% ± 0.8), and whole grain (15.2% ± 0.8). Similar patterns were noted when studied across age groups. The highest contribution from the enriched non-whole grain intake was among older adults at 70.9% ± 1.8 when compared to other grain subgroups, and the lowest contribution from the enriched non-whole group was among adolescents 8–13 years at 61.4% ± 2.1. Overall, in Canada the contribution of whole grains to grain products at snack occasions was significantly lower than the contribution of non-whole grains (enriched or not enriched).

## 4. Discussion

To our knowledge, this is the first study to examine the patterns of snack consumption among a representative sample of Canadians at the national and provincial levels. In all, 80.4% of the Canadian population reported consuming at least one snack per day, which varied between different age groups from 77.0% (≥55 years) to 96.4% (2–5 years). Among snack consumers, about 37% reported only one snack episode per day and nearly 10% reported four or more episodes of snacking per day. In general, children were more likely to have more snacking episodes than adolescents and adults. The percentage of total energy intake provided by snacking ranged from 20.8% to 27.0%, depending on the age group. There were no significant differences in obesity measures comparing snack consumers and non-consumers in children or adults. Moreover, Canadians obtained about one-quarter of their daily intake of milk and alternatives, and vegetables and fruit food groups from snacking.

A number of national surveys have been conducted in low- to middle-income and high-income countries to examine patterns of snack consumption among different age groups [9,11,21,22,23]. Our results revealed that the prevalence of snack consumption among Canadian adults aged 19–54 years was around 79%, which tended to be lower among older adults. Moreover, about 62% of adults aged 19–54 years and 58% of those ≥55 years reported two or more snacking episodes per day. In an analysis of cross-sectional data from the National Health and Nutrition Examination Survey (NHANES) conducted in 2003–2006, about 89% of American adults aged 20–74 years reported consuming a snack, and 66% and 63% of men and women, respectively, reported two or more snacking occasions per day [9]. From 2003–2006 to 2007–2010, American adults experienced a slight increase in the prevalence of snack consumption (from 89% to 90%), which was notably higher than our population [9]. In another study, snacking behaviors were examined cross-sectionally among a community sample of American working adults with a mean age of 43 years. Out of this sample population, nearly 85% reported at least one snacking episode per day [6]. However, the prevalence of snack consumption appears to be lower in low- to middle-income countries. In this regard, data from the first Brazilian nationally representative dietary survey conducted in 2008–2009 showed that the percentage of snacking among Brazilian adults ranged between 72.2% and 74.8%, depending on age subgroups [21]. Additionally, using four waves from the China Health and Nutrition Survey (CHNS), it was revealed that in spite of a marked transition in the snacking behaviors of Chinese between 1991 and 2009, only 35.6% of adults consumed a snack over a three-day period in 2009 [23].

In our population, the prevalence of snack consumption was considerably higher among children and adolescents than among adults. Our findings are in complete agreement with those obtained in other surveys [21,22,23]. More than 96% of younger children, aged 2–5 years, and about 93% of older children, aged 6-12 years, reported at least one episode of snacking per day, which was higher than that of adolescents (85.3%). Among snackers, about 83% of younger children, 80% of older children, and 75% of adolescents reported two or more snacking episodes per day. Using the most recent data from the NHANES (2011–2014), Dunford et al. found the highest prevalence of snack consumption among 2–5-year-old children (95%), followed by children aged 6–11 years (92%), and aged 12–18 years (83%), which are very close to our results. Moreover, the mean number of snacks consumed per day varied between 2.1 (younger children) and 3.0 (adolescents) [22]. Data from the Australian National Nutrition Survey conducted in 2011–2012 revealed that nearly 96% of children and adolescents (2–16 years) consumed at least one snack per day and over 80% reported consuming two or more snacks per day [11]. Similar to adults, children from lower-income countries were less likely to consume any snack. To support this claim, Duffey et al. showed that the percentage of Brazilian children and adolescents aged 10–18 years who reported snacking was 78.7% [21]. Moreover, according to CHNS, only 46.4%–58.8% of Chinese children and adolescents (2–18 years) consumed a snack over a three-day period in 2009, which was notably lower than Canadian children and adolescents [23].

Recent national surveys suggest that snacking is a major contributor to total energy intake, especially in the younger age groups. In our study, snacking contributed near 23% of total daily energy intake in the Canadian population. The percentage of daily energy intake from snacks was highest among younger children (27%) and lowest among older adults (20.8%). The total energy intake has relatively increased among American adults over the past decades, with shifts away from meals to snacks [9]. Based on results from the NHANES, snacking provided about 23% of 24-h energy intake for American adults in 2007–2010 [9]. Additionally, it was found that the percentage contribution of snacking to total energy intake was greater among American children, with approximately 25% of calories coming from snack occasions [24]. The percent contribution of snacking to total daily energy intake was reported to be 30.5% among Australian children and adolescents in 2011–2012, which experienced a substantial increase, from 24.1% in 1995 [11]. Among the Brazilian population aged ≥10 years, snacking accounted for 22% of total daily energy intake, which was closely similar to that observed in the Canadian population. However, in contrast to our observations, the highest percentage of daily energy intake from snacking was observed in older adults aged ≥60 years [21]. In China, the percentage of total energy intake provided by snacking in 2009 ranged from 12.3% in children aged 2–6 years to 4.0% in adults aged ≥19 years. The authors suggested that snacking on healthy food items including low-energy-density fruits rather than energy-dense, high-fat, salty or sweet foods may partly explain the observed differences between Chinese and other populations [23].

Inconsistent results have been reported regarding the association between snacking and BMI [3,25,26,27]. Our results showed no significant differences in obesity measures (i.e., BMI and BMI z-score) between snack consumers and non-consumers in both Canadian children and adults. These findings are similar to those of Hampl et al. who found that snacking patterns are associated with energy and nutrient intakes, but not with BMI among US adults [26]. In another study conducted on 4259 obese and 1092 reference Swedish men and women, Forslund and colleagues found no significant association between BMI and the number of snacks per day [25]. Hartmann et al. also found no association between snack frequency and BMI in a sample of 6189 adults participating in the Swiss Food Panel [7]. However, in contrast to these reports, Murakami and Livingstone reported that higher snack frequency was consistently associated with higher adiposity measures including BMI and waist circumference among British adults [3]. These inconsistent results were also observed among children and adolescents [4,27]. Evidence suggests that these inconsistent findings in the literature might be due to differences in snack food choices, which can vary from low-calorie nutrient-rich foods, such as fruits and vegetables, to high-calorie, nutrient-poor foods, such as desserts, cakes, and sugar-sweetened beverages [1,6].

In the current study, Canadians were more likely to select foods from milk and alternatives, and vegetables and fruit as snacks. Moreover, snack consumers tended to snack more on non-whole grain, enriched grain products. Hartmann et al. showed that the healthy cluster of high-frequency snack consumers was characterized by the highest consumption of fruit and vegetables and the lowest consumption frequency of unhealthy food groups such as sweets, savories, and meat. However, the unhealthy cluster was characterized by low consumption frequency of fruit and vegetables and the highest consumption of more unhealthy foods [7]. In another study, it was shown that among a sample of American working adults, the major source of energy from snacking was desserts and sweets (20.8%) followed by chips, crackers, ready-to-eat cereals, popcorn, and other related products (16.5%). In addition, dairy products and fruit and vegetables accounted for 15% and 12.5% of the calories from snacking, respectively [6]. Using the 1999–2002 NHANES, Zizza et al. reported that sugar, sweets, and beverages group were the major source of energy from snacking among American men and women with different food security categories. Grain products were the next largest energy source followed by the dairy group [28]. Furthermore, it has been reported that sweet snacks, savory pies, and coffee were three most frequently consumed snacks among Greek adults aged 20–50 years [29]. Considering the high frequency of unhealthy choices, Robinson and colleagues showed that messages about possible negative health effects of junk food and social normative messages can motivate young adults to reduce their consumption of high-calorie snack foods [30]. Furthermore, since snacking is a major contributor to total energy intake, it provides an opportunity to encourage people to snack more on nutrient-dense foods rather than high-calorie, nutrient-poor foods in order to improve overall diet quality.

### Strengths and Limitations

This study used the most recent nationally representative data to examine the patterns of snack consumption among different age groups, and thus the findings are generalizable to the Canadian population. Other strengths of the study include using measured, rather than self-reported BMI, and using the high-quality dietary assessment method (AMPM). We also acknowledge some limitations to our study. First, the absence of a universally accepted definition of snacking occasions often leads to difficulties in interpreting the results, particularly when comparing results across different studies [2,31]. Secondly, since CCHS is a cross-sectional survey, it only provides a descriptive snapshot of snacking patterns among the Canadian population at the national level and thus a causal direction between diet and health outcomes cannot be established. Thirdly, dietary intake was assessed using one 24-h recall, which is known to be memory dependent and may not reflect the usual intake. Moreover, people usually tend to underreport their dietary intake, especially when energy-dense, nutrient-poor foods are consumed as snacks [32,33].

## 5. Conclusions

In the present cross-sectional study using data from CCSH 2015, we showed that snacking is prevalent among the Canadians with over 80% reporting at least one snacking episode per day. Moreover, snacking is an important contributor to total energy intake in the Canadian population, especially among the younger age groups. Snacking also contributed significantly to micronutrient intakes, especially vitamin C and potassium. Our findings demonstrated no significant association between snacking behavior and BMI among children and adults.

## Figures and Tables

**Table 1:** Sociodemographic characteristics of Canadian children (2–18 years) and adults (19+ years) as snack consumers and non-consumers ^1^.

Characteristic	Children/Teens (2–18 years) *n* = 6,430,741		Adults (≥19 years) *n* = 27,008,078	
	Snack (Mean or %, SE) *n* = 5,817,816	No Snack (Mean or %, SE) *n* = 612,925	Snack (Mean or %, SE) *n* = 21,078,984	No Snack (Mean or %, SE) *n* = 5,929,094
**Mean age +/− SD (y)**	9.6 ± 0.1	12.2 ± 0.3 *	48.9 ± 0.2	50.5 ± 0.6 *
**Sex (% male)**	50.3 (0.8)	48.2 (3.4)	49.5 (0.4)	51.0 (1.6)
**Smoker (% yes) ^2^**	3.3 (0.5)	4.2 (1.2)	18.6 (0.8)	19.5 (1.4)
**Ethnicity (% Caucasian)**	68.6 (1.4)	56.9 (3.9)*	75.9 (1.02)	71.8 (1.9) *
**Education (% university grad) ^3^**	45.0 (1.2)	39.4 (3.5)	39.5 (1.03)	34.6 (1.9) *
**Marital status (% married or co-habiting)**	N/A	N/A	64.8 (0.9)	61.1 (1.8)
**Food secure (% yes)**	83.8 (0.9)	85.7 (2.3)	88.1 (0.6)	90.2 (1.01)
**BMI (kg/m^2^)**	N/A	N/A	27.3 (0.1)	27.7 (0.2)
**BMI z-score ^4^**	0.46 (0.04)	0.44 (0.1)	N/A	N/A
**Overweight/obese (% yes)**	26.1 (1.1)	27.5 (3.7)	62.2 (1.2)	60.5 (2.3)
**Urban residence (% yes)**	81.7 (1.1)	86.8 (2.2)	82.3 (0.9)	83.3 (1.3)
**Immigrant to Canada (% yes) ^5^**	8.5 (0.6)	14.4 (2.4)	27.8 (1.1)	26.1 (1.7)

Data source: 2015 Canadian Community Health Survey–Nutrition. Any individual who reported consuming snack as a food occasion on day 1 of 24-h recall was defined as a snack consumer. * Significant at 0.05 level of significance using a chi-squared test for categorical variables and t-test for continuous variables. Snack consumers were compared to snack non-eaters separately for children and adults. ^1^ All data were weighted and bootstrapped to the Canadian population; ^2^ for children, data are presented as prevalence among those aged ≥12 years, as smoking was queried only in this age group; ^3^ for children, these data reflect whether a member of the household is or is not a university graduate; ^4^ for those aged 5–18 years, based on body mass index (BMI) z-score for age; ^5^ note that the desired data here are simply the proportion who are immigrants to Canada.

**Table 2:** Percent contribution of nutrients from food occasions to daily intake among snack consumers (*n* = 26,896,800) ^1^.

Nutrient	Snack	Breakfast/Brunch	Lunch	Dinner
	% ± SE	% ± SE	% ± SE	% ± SE
**Energy (%)**	22.7 ± 0.3	19.2 ± 0.2 ^a^	22.8 ± 0.2	31.3 ± 0.2 ^c^
**Carbohydrates (%)**	25.8 ± 0.3	21.9 ± 0.2 ^a^	21.1 ± 0.2 ^b^	26.7 ± 0.3 ^c^
**Dietary fibers (%)**	25.8 ± 0.3	22.1 ± 0.3 ^a^	22.4 ± 0.3 ^b^	28.4 ± 0.3 ^c^
**Total sugars (%)**	33.0 ± 0.4	23.5 ± 0.3 ^a^	16.9 ± 0.3 ^b^	19.4 ± 0.3 ^c^
**Total Fat (%)**	23.4 ± 0.3	17.9 ± 0.3 ^a^	24.2 ± 0.3 ^b^	32.5 ± 0.3 ^c^
**SFA (%)**	22.9 ± 0.3	19.2 ± 0.3 ^a^	23.6 ± 0.3	31.6 ± 0.3 ^c^
**MUFA (%)**	23.4 ± 0.3	17.2 ± 0.3 ^a^	24.1 ± 0.3 ^b^	33.5 ± 0.3 ^c^
**PUFA (%)**	23.0 ± 0.4	17.5 ± 0.3 ^a^	25.5 ± 0.3 ^b^	32.9 ± 0.3 ^c^
**Cholesterol (%)**	12.4 ± 0.3	20.1 ± 0.4 ^a^	25.8 ± 0.4 ^b^	39.5 ± 0.4 ^c^
**Protein (%)**	14.7 ± 0.2	18.0 ± 0.2 ^a^	26.1 ± 0.2 ^b^	38.8 ± 0.3 ^c^
**Vitamin A RAE (%)**	16.8 ± 0.3	23.6 ± 0.4 ^a^	23.0 ± 0.4 ^b^	32.7 ± 0.4 ^c^
**Vitamin D (%)**	14.4 ± 0.4	35.3 ± 0.5 ^a^	19.1 ± 0.5 ^b^	25.9 ± 0.4 ^c^
**Vitamin C (%)**	23.4 ± 0.5	16.3 ± 0.4 ^a^	23.0 ± 0.4 ^b^	32.5 ± 0.5 ^c^
**Thiamin (%)**	16.7 ± 0.3	24.5 ± 0.3 ^a^	23.8 ± 0.3 ^b^	32.0 ± 0.3 ^c^
**Riboflavin (%)**	18.4 ± 0.3	26.9 ± 0.3 ^a^	20.5 ± 0.3 ^b^	27.3 ± 0.3 ^c^
**Niacin NEA (%)**	14.7 ± 0.2	17.9 ± 0.2 ^a^	25.7 ± 0.2 ^b^	38.9 ± 0.3 ^c^
**Vitamin B_6_ (%)**	17.6 ± 0.3	18.1 ± 0.3	23.2 ± 0.3 ^b^	37.8 ± 0.3 ^c^
**Vitamin B_12_ (%)**	13.4 ± 0.3	23.7 ± 0.4 ^a^	23.3 ± 0.4 ^b^	35.1 ± 0.4 ^c^
**Folic acid (%)**	17.4 ± 0.4	27.9 ± 0.5 ^a^	25.3 ± 0.5 ^b^	28.6 ± 0.5 ^c^
**Folate DFE (%)**	18.0 ± 0.3	22.2 ± 0.3 ^a^	24.6 ± 0.3 ^b^	32.2 ± 0.3 ^c^
**Calcium (%)**	21.1 ± 0.3	25.9 ± 0.3 ^a^	20.8 ± 0.3	25.0 ± 0.3 ^c^
**Magnesium (%)**	21.2 ± 0.3	22.5 ± 0.3 ^a^	20.9 ± 0.3	28.5 ± 0.3 ^c^
**Iron (%)**	18.2 ± 0.3	24.1 ± 0.3 ^a^	23.4 ± 0.3 ^b^	32.1 ± 0.3 ^c^
**Zinc (%)**	16.4 ± 0.3	19.5 ± 0.3 ^a^	24.7 ± 0.3 ^b^	36.7 ± 0.3 ^c^
**Sodium (%)**	14.8 ± 0.3	17.6 ± 0.3 ^a^	28.9 ± 0.3 ^b^	36.3 ± 0.3 ^c^
**Potassium (%)**	20.7 ± 0.3	20.0 ± 0.2 ^a^	21.6 ± 0.2	31.8 ± 0.3 ^c^

Data source: 2015 Canadian Community Health Survey–Nutrition. ^1^ All data were weighted and bootstrapped to the Canadian population. DFE = dietary folate equivalents, NEA = niacin equivalents, RAE = retinol activity equivalents. a = significant difference between snack and breakfast/brunch consumption, b = significant difference between snack and lunch consumption, c = significant difference between snack and dinner consumption at 5% level of significance.

**Table 3:** Relative contribution of snacks to daily energy and nutrient intake across the five age groups among snack consumers ^1^.

Nutrients	2–5 years % ± SE	6–12 years % ± SE	13–18 years % ± SE	19–54 years % ± SE	≥55 years % ± SE
**Energy (%)**	27.0 ± 0.6	26.5 ± 0.5	24.9 ± 0.6	22.3 ± 0.5	20.8 ± 0.4 *
**Carbohydrates (%)**	29.9 ± 0.7	29.8 ± 0.6	28.1 ± 0.5	25.3 ± 0.5	24.2 ± 0.5 *
**Dietary fibers (%)**	30.4 ± 0.9	29.6 ± 0.7	29.3 ± 0.6	25.9 ± 0.6	22.9 ± 0.5 *
**Total sugars (%)**	37.0 ± 0.9	35.8 ± 0.7	34.0 ± 0.7	32.8 ± 0.7	31.7 ± 0.6 *
**Total Fat (%)**	27.2 ± 1.0	26.4 ± 0.7	25.1 ± 0.7	23.5 ± 0.6	21.4 ± 0.5 *
**SFA (%)**	26.8 ± 1.1	25.9 ± 0.7	24.7 ± 0.8	22.9 ± 0.6	21.2 ± 0.5 *
**MUFA (%)**	26.6 ± 1.1	25.8 ± 0.7	24.9 ± 0.8	23.6 ± 0.6	21.4 ± 0.5 *
**PUFA (%)**	27.8 ± 1.0	26.7 ± 0.8	24.6 ± 0.8	23.3 ± 0.7	20.2 ± 0.5 *
**Cholesterol (%)**	17.4 ± 0.9	14.8 ± 0.6	14.2 ± 0.6	11.8 ± 0.5	11.5 ± 0.4 *
**Protein (%)**	19.6 ± 0.7	17.0 ± 0.5	16.5 ± 0.5	14.6 ± 0.4	13.1 ± 0.3 *
**Vitamin A RAE (%)**	20.0 ± 0.9	20.0 ± 0.7	19.7 ± 0.8	16.6 ± 0.6	15.0 ± 0.5 *
**Vitamin D (%)**	17.7 ± 0.6	16.9 ± 0.5	15.6 ± 0.6	14.8 ± 0.5	12.4 ± 0.4 *
**Vitamin C (%)**	30.2 ± 1.7	29.7 ± 1.0	25.8 ± 0.9	22.9 ± 0.8	20.5 ± 0.7 *
**Thiamin (%)**	20.4 ± 0.7	19.2 ± 0.6	18.8 ± 0.6	16.8 ± 0.4	14.8 ± 0.4 *
**Riboflavin (%)**	24.3 ± 0.9	21.2 ± 0.5	20.1 ± 0.6	18.1 ± 0.5	16.8 ± 0.3 *
**Niacin NEA (%)**	18.7 ± 0.6	16.8 ± 0.5	16.6 ± 0.5	14.6 ± 0.4	13.1 ± 0.3
**Vitamin B_6_ (%)**	22.6 ± 0.8	20.8 ± 0.6	20.3 ± 0.6	17.6 ± 0.5	15.3 ± 0.4 *
**Vitamin B_12_ (%)**	18.4 ± 0.9	16.1 ± 0.6	15.9 ± 0.7	13.1 ± 0.5	11.7 ± 0.4 *
**Folic acid (%)**	20.3 ± 1.2	19.2 ± 0.8	18.3 ± 0.8	16.7 ± 0.6	17.1 ± 0.7 *
**Folate DFE (%)**	21.4 ± 0.9	20.7 ± 0.6	19.4 ± 0.6	17.6 ± 0.5	16.9 ± 0.4 *
**Calcium (%)**	26.3 ± 1.1	23.3 ± 0.6	22.0 ± 0.7	20.9 ± 0.6	19.7 ± 0.5 *
**Magnesium (%)**	24.9 ± 0.7	23.9 ± 0.5	23.9 ± 0.6	21.3 ± 0.5	19.1 ± 0.4 *
**Iron (%)**	21.9 ± 0.8	21.3 ± 0.5	21.5 ± 0.6	18.2 ± 0.5	15.9 ± 0.4 *
**Zinc (%)**	20.5 ± 0.7	18.4 ± 0.5	18.6 ± 0.6	16.5 ± 0.5	14.3 ± 0.3 *
**Sodium (%)**	18.6 ± 0.7	18.1 ± 0.5	17.6 ± 0.6	14.8 ± 0.5	12.7 ± 0.4 *
**Potassium (%)**	25.7 ± 0.7	24.5 ± 0.5	23.7 ± 0.6	20.7 ± 0.5	18.2 ± 0.4 *

Data source: 2015 Canadian Community Health Survey–Nutrition. ^1^ All data were weighted and bootstrapped to the Canadian population. DFE = dietary folate equivalents, NE = niacin equivalents, RAE = retinol activity equivalents. * Significant difference among the age groups at 5% level of significance. Weighted frequency of age groups: 2–5 years: *n* = 1,181,823; 6–12 years: *n* = 2,407,637; 13–18 years: *n* = 2,228,356; 19–54 years: *n* = 12,768,195; 55+ years: *n* = 8,310,789.

**Table 4:** Percent contribution of whole grain and non-whole grain (enriched and not enriched) to total grain products at snack occasion ^1^.

	Whole Grain	Non-Whole Grain, Enriched	Non-Whole Grain, Not Enriched
**Age groups**	%± SE	% ± SE	% ± SE
**All ages (≥2 years) *n* = 26,896,800**	15.2 ± 0.8	66.6 ± 1.0	18.2 ± 0.8 *
**2–5 years *n* = 1,181,823**	15.6 ± 2.2	69.2 ± 2.8	15.2 ± 2.0 *
**6–12 years *n* = 2,407,637**	15.8 ± 1.4	62.6 ± 2.0	21.5 ± 1.9 *
**13–18 years *n* = 2,228,356**	15.0 ± 1.8	61.4 ± 2.1	23.6 ± 1.8 *
**19–54 years *n* = 12,768,195**	14.6 ± 1.3	65.8 ± 1.8	19.7 ± 1.4 *
**≥55 years *n* = 8,310,789**	15.9 ± 1.5	70.9 ± 1.8	13.2 ± 1.4 *

Data source: 2015 Canadian Community Health Survey–Nutrition. ^1^ All data were weighted and bootstrapped to the Canadian population. * Significant difference between contribution of various grain groups to snack occasion at 5% level of significance.

## Abbreviations

Polyunsaturated fatty acids (PUFA); monounsaturated fatty acids (MUFA); saturated fatty acids (SFA); Canadian Community Health Survey (CCHS); body mass index (BMI); National Diet and Nutrition Survey (NDNS); Automated Multiple Pass Method (AMPM); World Health Organization (WHO); National Health and Nutrition Examination Survey (NHANES); China Health and Nutrition Survey (CHNS).

## References

[1] Bellisle F. (2014). Meals and snacking, diet quality and energy balance. Physiol. Behav..

[2] Johnson G.H., Anderson G.H. (2010). Snacking definitions: Impact on interpretation of the literature and dietary recommendations. Crit. Rev. Food Sci. Nutr..

[3] Murakami K., Livingstone M.B. (2016). Associations between meal and snack frequency and diet quality and adiposity measures in British adults: Findings from the National Diet and Nutrition Survey. Public Health Nutr..

[4] Murakami K., Livingstone M.B. (2016). Associations between meal and snack frequency and overweight and abdominal obesity in US children and adolescents from National Health and Nutrition Examination Survey (NHANES) 2003–2012. Br. J. Nutr..

[5] Na L., Han T., Zhang W., Wu X., Na G., Du S., Sun C. (2015). A Snack Dietary Pattern Increases the Risk of Hypercholesterolemia in Northern Chinese Adults: A Prospective Cohort Study. PLoS ONE.

[6] Barnes T.L., French S.A., Harnack L.J., Mitchell N.R., Wolfson J. (2015). Snacking behaviors, diet quality, and body mass index in a community sample of working adults. J. Acad. Nutr. Diet..

[7] Hartmann C., Siegrist M., van der Horst K. (2013). Snack frequency: Associations with healthy and unhealthy food choices. Public Health Nutr..

[8] Maugeri A., Kunzova S., Medina-Inojosa J.R., Agodi A., Barchitta M., Homolka M., Kiacova N., Bauerova H., Sochor O., Lopez-Jimenez F. (2018). Association between eating time interval and frequency with ideal cardiovascular health: Results from a random sample Czech urban population. Nutr. Metab. Cardiovasc. Dis..

[9] Kant A.K., Graubard B.I. (2015). 40-year trends in meal and snack eating behaviors of American adults. J. Acad. Nutr. Diet..

[10] Nielsen S.J., Siega-Riz A.M., Popkin B.M. (2002). Trends in energy intake in U.S. between 1977 and 1996: Similar shifts seen across age groups. Obes. Res..

[11] Fayet-Moore F., Peters V., McConnell A., Petocz P., Eldridge A.L. (2017). Weekday snacking prevalence, frequency, and energy contribution have increased while foods consumed during snacking have shifted among Australian children and adolescents: 1995, 2007 and 2011–12 National Nutrition Surveys. Nutr. J..

[12] Kerr M.A., Rennie K.L., McCaffrey T.A., Wallace J.M., Hannon-Fletcher M.P., Livingstone M.B. (2009). Snacking patterns among adolescents: A comparison of type, frequency and portion size between Britain in 1997 and Northern Ireland in 2005. Br. J. Nutr..

[13] Piernas C., Popkin B.M. (2010). Snacking increased among U.S. adults between 1977 and 2006. J. Nutr..

[14] Gilbert J.A., Miller D., Olson S., St-Pierre S. (2012). After-school snack intake among Canadian children and adolescents. Can. J. Public Health.

[15] Canadian Community Health Survey (CCHS): Nutrition-2015 (First Interview) (2017). http://www23.statcan.gc.ca/imdb/p3Instr.pl?Function=assembleInstr&lang=en&Item_Id=202664.

[16] (2017). Canadian Community Health Survey-Nutrition (CCHS). http://www23.statcan.gc.ca/imdb/p2SV.pl?Function=getSurvey&SDDS=5049#a1.

[17] Canada Food Guide (2007). Eating Well with Canada Food Guide. https://www.canada.ca/content/dam/hc-sc/migration/hc-sc/fn-an/alt_formats/hpfb-dgpsa/pdf/food-guide-aliment/print_eatwell_bienmang-eng.pdf.

[18] Canada’s Food Guide (2019). Government of Canada. https://food-guide.canada.ca/en/.

[19] World Health Organization (2011). WHO Anthro (Version 3.2.2, January 2011) and Macros. https://www.who.int/childgrowth/software/en/).

[20] Statistics Canada (2014). The Research Data Centres Information and Technical Bulletin—Weighted Estimation and Bootstrap Variance Estimation for Analyzing Survey Data: How to Implement in Selected Software. http://www5.statcan.gc.ca/olc-cel/olc?ObjId=12-002-X201400111901&ObjType=47&lang=en.

[21] Duffey K.J., Pereira R.A., Popkin B.M. (2013). Prevalence and energy intake from snacking in Brazil: Analysis of the first nationwide individual survey. Eur. J. Clin. Nutr..

[22] Dunford E.K., Popkin B.M. (2018). 37 year snacking trends for US children 1977–2014. Pediatr. Obes..

[23] Wang Z., Zhai F., Zhang B., Popkin B.M. (2012). Trends in Chinese snacking behaviors and patterns and the social-demographic role between 1991 and 2009. Asia Pac. J. Clin. Nutr..

[24] Wang D., van der Horst K., Jacquier E., Eldridge A.L. (2016). Snacking Among US Children: Patterns Differ by Time of Day. J. Nutr. Educ. Behav..

[25] Berteus Forslund H., Torgerson J.S., Sjostrom L., Lindroos A.K. (2005). Snacking frequency in relation to energy intake and food choices in obese men and women compared to a reference population. Int. J. Obes. (Lond.).

[26] Hampl J.S., Heaton C.L., Taylor C.A. (2003). Snacking patterns influence energy and nutrient intakes but not body mass index. J. Hum. Nutr. Diet..

[27] Keast D.R., Nicklas T.A., O’Neil C.E. (2010). Snacking is associated with reduced risk of overweight and reduced abdominal obesity in adolescents: National Health and Nutrition Examination Survey (NHANES) 1999–2004. Am. J. Clin. Nutr..

[28] Zizza C.A., Duffy P.A., Gerrior S.A. (2008). Food insecurity is not associated with lower energy intakes. Obesity (Silver Spring).

[29] Elena F., Maria B. (2006). Snack patterns of Greek adults 20–50 years of age. J. Foodserv..

[30] Robinson E., Harris E., Thomas J., Aveyard P., Higgs S. (2013). Reducing high calorie snack food in young adults: A role for social norms and health based messages. Int. J. Behav. Nutr. Phys. Act..

[31] Chamontin A., Pretzer G., Booth D.A. (2003). Ambiguity of ‘snack’ in British usage. Appetite.

[32] Champagne C.M., Baker N.B., DeLany J.P., Harsha D.W., Bray G.A. (1998). Assessment of energy intake underreporting by doubly labeled water and observations on reported nutrient intakes in children. J. Am. Diet. Assoc..

[33] Poslusna K., Ruprich J., de Vries J.H., Jakubikova M., van’t Veer P. (2009). Misreporting of energy and micronutrient intake estimated by food records and 24 hour recalls, control and adjustment methods in practice. Br. J. Nutr..

